# Bromido[1-(η^6^-4-*tert*-butyl­benz­yl)-3-(2,4,6-trimethyl­benz­yl)benzimidazol-2-yl­idene]chloridoruthenium(II)

**DOI:** 10.1107/S1600536808042256

**Published:** 2008-12-20

**Authors:** Hakan Arslan, Don VanDerveer, İsmail Özdemir, Serpil Demir, Bekir Çetinkaya

**Affiliations:** a Department of Natural Sciences, Fayetteville State University, Fayetteville, NC 28301, USA; b Department of Chemistry, Faculty of Pharmacy, Mersin University, Mersin, TR 33169, Turkey; c Department of Chemistry, Clemson University, Clemson, SC 29634, USA; d Department of Chemistry, Faculty of Science and Arts, İnönü University, Malatya, TR 44280, Turkey; e Department of Chemistry, Faculty of Science, Ege University, Bornova-İzmir, TR 35100, Turkey

## Abstract

A new ruthenium complex, [RuBrCl(C_28_H_32_N_2_)], has been synthesized and characterized by elemental analysis, ^1^H NMR, ^13^C NMR, IR-spectroscopy and a single-crystal X-ray diffraction study. The Ru atom in this complex is best described as having a considerably distorted octa­hedral coordination environment with the arene occupying three coordination sites. Two further coordination sites are occupied by chloride and bromide ligands, while the sixth site is occupied by the carbene. The carbene portion of the ligand is a benzimidazole ring. This ring is connected to the C_6_H_4_C(CH_3_)_3_ arene by a CH_2_ bridge. This leads to a system with very little apparent strain. The two halogen atoms are disordered between Br and Cl. Two partial Cl atoms share the same sites as two partial Br atoms so that the title compound effectively has one Cl and one Br atom. C—H⋯*X* (*X* = Cl, Br) hydrogen bonds help to stabilize the crystal structure.

## Related literature

For synthesis, see: Yaşar *et al.* (2008 ▶); Çetinkaya *et al.* (2003 ▶). For general background, see: Herrmann (2002 ▶); Arduengo & Krafczyc (1998 ▶); Arduengo *et al.* (1991 ▶). For related compounds, see: Begley *et al.* (1991 ▶); Arslan *et al.* (2004*b* ▶, 2005*a* ▶,*b* ▶, 2007*b* ▶,*c* ▶). For related literature, see: Arslan *et al.* (2004*a* ▶, 2007*a* ▶); Herrmann *et al.* (1995 ▶); Navarro *et al.* (2006 ▶); Özdemir *et al.* (2001 ▶); Çetinkaya *et al.* (2001 ▶, 2002 ▶).
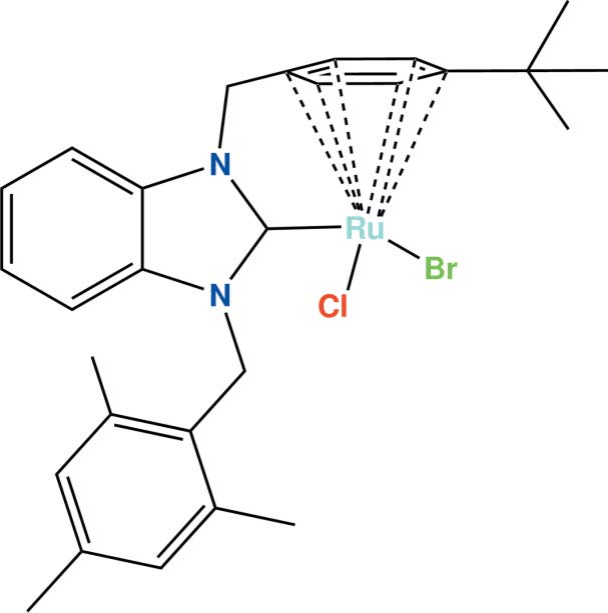


## Experimental

### 

#### Crystal data

[RuBrCl(C_28_H_32_N_2_)]
*M* *_r_* = 611.69
Monoclinic, 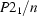

*a* = 7.6336 (15) Å
*b* = 27.725 (6) Å
*c* = 12.051 (2) Å
β = 98.80 (3)°
*V* = 2520.5 (9) Å^3^
*Z* = 4
Mo *K*α radiation
μ = 2.29 mm^−1^
*T* = 153 (2) K
0.26 × 0.12 × 0.02 mm

#### Data collection

Rigaku Mercury CCD diffractometer
Absorption correction: multi-scan (*REQAB*; Jacobson, 1998 ▶) *T*_min_ = 0.581, *T*_max_ = 0.955
17799 measured reflections
4449 independent reflections
3288 reflections with *I* > 2σ(*I*)
*R*_int_ = 0.089

#### Refinement

*R*[*F* ^2^ > 2σ(*F* ^2^)] = 0.089
*wR*(*F* ^2^) = 0.255
*S* = 1.04
4449 reflections
306 parameters
H-atom parameters constrained
Δρ_max_ = 2.45 e Å^−3^
Δρ_min_ = −1.38 e Å^−3^

### 

Data collection: *CrystalClear* (Rigaku/MSC, 2006 ▶); cell refinement: *CrystalClear*; data reduction: *CrystalClear*; program(s) used to solve structure: *SHELXTL* (Sheldrick, 2008 ▶); program(s) used to refine structure: *SHELXTL*; molecular graphics: *SHELXTL*; software used to prepare material for publication: *SHELXTL*.

## Supplementary Material

Crystal structure: contains datablocks global, I. DOI: 10.1107/S1600536808042256/at2686sup1.cif

Structure factors: contains datablocks I. DOI: 10.1107/S1600536808042256/at2686Isup2.hkl

Additional supplementary materials:  crystallographic information; 3D view; checkCIF report

## Figures and Tables

**Table 1:** Hydrogen-bond geometry (Å, °)

*D*—H⋯*A*	*D*—H	H⋯*A*	*D*⋯*A*	*D*—H⋯*A*
C14—H14⋯Cl2^i^	0.96	2.65	3.406 (11)	135
C16—H16*C*⋯Br1	0.96	2.92	3.563 (11)	125
C19—H19*A*⋯Br1	0.96	2.92	3.323 (11)	106

Symmetry code: (i) 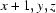.

**Fig. 1.**
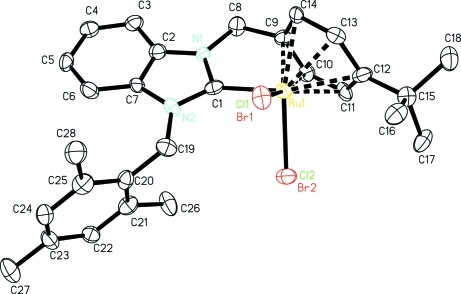
The molecular structure of the title compound, showing the atom-numbering scheme and displacement ellipsoids drawn at the 50% probability level.

**Fig. 2.**
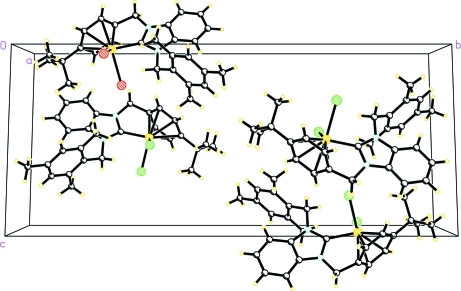
A packing diagram for (I).

**Fig. 3.**
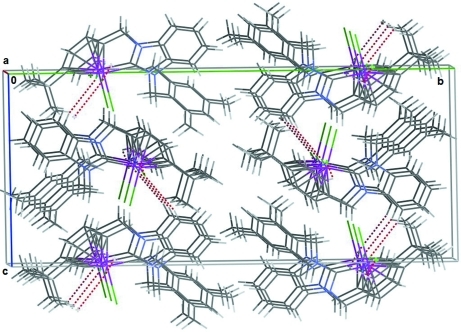
A view of the packing diagram of (I). Hydrogen bonds are shown as dashed lines.

**Fig. 4.**
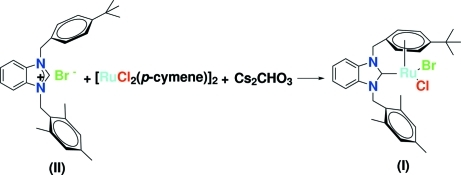
Preparation of the title compound.

## supplementary crystallographic information

### Comment

*N*-Heterocyclic carbenes have received great attention since the first synthesis of a carbene compound,1,3-di-l-adamantylimidazol-2-ylidene, by Arduengo *et al.*, (1991). *N*-heterocyclic carbenes generally derived from imidazolium, tetrahydropyrimidin-1-ium and benzimidazolium salts, have attracted wide spread attention as ligands for main group elements and transition metals. The *N*-heterocyclic carbene metal complexes are remarkably stable toward heat, air, and moisture, and many organic reactions using these complexes as catalysts have been investigated. These include Suzuki-Miyura, Sonogashira, Stille and Heck reactions (Herrmann *et al.*, 1995; Herrmann, 2002; Navarro *et al.*, 2006; Arduengo & Krafczyc,1998).

Previous work from our research groups in this area has focused on the elaboration of olefins as electron-rich heterocyclic carbene precursors to allow the formation of chelating carbenes. We have also looked at the rapidly developing chemistry of *η*^6^-arene ruthenium(II) complexes containing substituted imidazolidin-2-ylidenes (Özdemir *et al.*, 2001; Çetinkaya *et al.*, 2001, 2002, 2003), and on synthesis, characterization, crystal structure, and using palladium, platinum and ruthenium *N*-heterocyclic carbene complexes as catalysts (Yaşar *et al.*, 2008; Arslan *et al.*, 2007*a*, 2007*b*, 2007*c*, 2004*a*, 2004*b*, 2005*a*, 2005*b*).

In the present study, we have synthesized and characterized a new ruthenium complex, (1-(4-*tert*-butylbenzyl)-3-(2,4,6-trimethylbenzyl)-benzimidazol-2-ylidene)ruthenium(II) bromide chloride, (I). The molecular structure of the title compound, (I), is depicted in Fig. 1.

The carbene portion of the ligand is a benzimidazole ring. This ring is connected to the C_6_H_4_C(CH_3_)_3_ arene by a CH_2_ bridge. This leads to a system with very little apparent strain. The ruthenium atom in the title compound is best described as having an octahedral coordination environment, with the arene occupying three coordination sites. Two further coordination sites are occupied by Cl and Br ligands, while the sixth site is occupied by the carbene carbon of benzimidazole ring. The ruthenium atom is situated 1.658 (5) Å from the ring centroid of the arene atom. While there are substantial differences in the C—C and C—Ru distances for the arene ring, there is no evidence of the alternating C—C bonds observed in some ruthenium-arene complexes (Begley *et al.*, 1991). The arene ring is essentially planar, the mean deviation from the plane being 0.013 (12) Å. In addition, the trimethylbenzyl and benzimidazole rings are almost planar with the maximum deviations of 0.009 (12) Å for atom C25 and, 0.003 (11) Å for atom C2. The benzimidazole moiety is planar and it forms dihedral angles of 87.16 (6)° and 85.31 (7)°, respectively, with the mean planes through the arene and trimethylbenzyl rings.

The two halogen atoms are disordered between Br and Cl. Two chlorine atoms share the same site as two bromine atoms so that the title compound effectively has one Cl and one Br atom. The occupancies of the Cl1 and Cl2 atoms are 0.399 (11) and 0.630 (11), respectively, and those of the Br1 and Br2 atoms are 0.601 (11) and 0.370 (11), respectively.

The structure of the title compound is assembled by intermolecular C—H···Cl hydrogen bonds, to form a two-dimensional framework (Fig. 2, 3 and Table 1) (Macrae *et al.*, 2006). The intermolecular contacts, C—H···Br, are also listed in Table 1.

### Experimental

All reactions for the preparation of (I) and (II) were carried out under Ar in flame-dried glass-ware using standard Schlenk-type flasks. The solvents used were purified by distillation over the drying agents indicated and were transferred under Ar: CH_2_Cl_2_ (P_4_O_10_), hexane, toluene (Na). RuClBr[η^1^-CN{CH_2_(η^6^-C_6_H_4_CMe_3_-4)}C_6_H_4_N(CH_2_C_6_H_2_Me_3_-2,4,6)]: A suspension of 1-(4-terbutylbenzyl)-3-(2,4,6-trimethylbenzyl)benzimidazolium bromide (1.00 g, 2.10 mmol), Cs_2_CO_3_ (0.7 g, 2.14 mmol), [RuCl_2_(*p*-cymene)]_2_ (0.5 g, 0.82 mmol) and molecular sieves was heated under reflux in degassed dry toluene (20 ml) for 12 h. The reaction mixture was then filtered while hot, and the volume was reduced to about 10 ml before addition of *n*-hexane (10 ml). The precipitate formed was crystallized from CH_2_Cl_2_/hexane (5:10 ml) to give the crystal product (Fig. 3). Yield 1.00 g (82%). *M*.p.: 339–340 *^o^*C. FT—IR (KBr pellet, cm^-1^): ν_CN_ 1432 cm^-1^. Anal. Found: C, 54.91; H, 5.29; N: 4.54. Calc. for C_28_H_32_N_2_RuClBr: C, 54.86; H, 5.26; N, 4.57. ^1^H NMR (δ, 399.9 MHz, CDCl_3_): 1.25, 1.31 and 1.37 [s, 9H, CH_2_C_6_H_4_C(C*H*_3_)_3_-*p*]; 2.17 [m, 9H, CH_2_C_6_H_2_(C*H*_3_)_3_-2,4,6]; 5.07 and 5.67 [m, 4H, C*H*_2_C_6_H_2_(CH_3_)_3_-2,4,6 and C*H*_2_C_6_H_4_C(CH_3_)_3_-*p*]; 6.79–7.60 [m, 10H, NC6*H*4 N, CH_2_C_6_*H*_2_(CH_3_)_3_-2,4,6 and CH_2_C_6_*H*_4_C(CH_3_)_3_-*p*]. ^13^C {H} NMR (δ, 100.5 MHz, CDCl_3_): 20.4 and 21.5 [CH_2_C_6_H_2_(*C*H_3_)_3_-2,4,6]; 31.3 [CH_2_C_6_H_4_C(*C*H_3_)_3_-*p*]; 34.7 [CH_2_C_6_H_4_*C*(CH_3_)_3_-*p*]; 45.0 [*C*H_2_C_6_H_2_(CH_3_)_3_-2,4,6]; 53.2 [*C*H_2_C_6_H_4_C(CH_3_)_3_-*p*]; 89.1, 90.1, 91.1, 93.0, 93.7, 98.9, 100.9, 109.7, 113.0, 113.8, 123.3, 125.3, 127.9, 129.0, 131.5, 133.4, 137.9 and 150.1 [CH_2_*C*_6_H_2_(CH_3_)_3_-2,4,6;N*C*_6_H_4_N and CH_2_*C*_6_H_4_C(CH_3_)_3_-*p*]; 184.1 [*C*_carbene_].

### Crystal data

**Table:**

[RuBrCl(C_28_H_32_N_2_)]	*F*(000) = 1237.9
*M**_r_* = 611.69	*D*_x_ = 1.612 Mg m^−^^3^
Monoclinic, *P*2_1_/*n*	Mo *K*α radiation, λ = 0.71073 Å
Hall symbol: -P 2yn	Cell parameters from 6078 reflections
*a* = 7.6336 (15) Å	θ = 2.8–26.3°
*b* = 27.725 (6) Å	µ = 2.29 mm^−^^1^
*c* = 12.051 (2) Å	*T* = 153 K
β = 98.80 (3)°	Plate, red
*V* = 2520.5 (9) Å^3^	0.26 × 0.12 × 0.02 mm
*Z* = 4	

### Data collection

**Table:**

Rigaku Mercury CCD diffractometer	4449 independent reflections
Radiation source: Sealed Tube	3288 reflections with *I* > 2σ(*I*)
Graphite Monochromator	*R*_int_ = 0.089
Detector resolution: 14.6306 pixels mm^-1^	θ_max_ = 25.0°, θ_min_ = 2.3°
ω scans	*h* = −9→9
Absorption correction: multi-scan (REQAB; Jacobson, 1998)	*k* = −30→33
*T*_min_ = 0.581, *T*_max_ = 0.955	*l* = −14→14
17799 measured reflections	

### Refinement

**Table:**

Refinement on *F*^2^	Primary atom site location: structure-invariant direct methods
Least-squares matrix: full	Secondary atom site location: difference Fourier map
*R*[*F*^2^ > 2σ(*F*^2^)] = 0.089	Hydrogen site location: inferred from neighbouring sites
*wR*(*F*^2^) = 0.255	H-atom parameters constrained
*S* = 1.04	*w* = 1/[σ^2^(*F*_o_^2^) + (0.1351*P*)^2^ + 37.3742*P*] where *P* = (*F*_o_^2^ + 2*F*_c_^2^)/3
4449 reflections	(Δ/σ)_max_ < 0.001
306 parameters	Δρ_max_ = 2.45 e Å^−^^3^
0 restraints	Δρ_min_ = −1.38 e Å^−^^3^

### Special details

**Table:**

Geometry. All e.s.d.'s (except the e.s.d. in the dihedral angle between two l.s. planes) are estimated using the full covariance matrix. The cell e.s.d.'s are taken into account individually in the estimation of e.s.d.'s in distances, angles and torsion angles; correlations between e.s.d.'s in cell parameters are only used when they are defined by crystal symmetry. An approximate (isotropic) treatment of cell e.s.d.'s is used for estimating e.s.d.'s involving l.s. planes.
Refinement. Refinement of *F*^2^ against ALL reflections. The weighted *R*-factor *wR* and goodness of fit *S* are based on *F*^2^, conventional *R*-factors *R* are based on *F*, with *F* set to zero for negative *F*^2^. The threshold expression of *F*^2^ > σ(*F*^2^) is used only for calculating *R*-factors(gt) *etc*. and is not relevant to the choice of reflections for refinement. *R*-factors based on *F*^2^ are statistically about twice as large as those based on *F*, and *R*- factors based on ALL data will be even larger.

### Fractional atomic coordinates and isotropic or equivalent isotropic displacement parameters (Å^2^)

**Table:**

	*x*	*y*	*z*	*U*_iso_*/*U*_eq_	Occ. (<1)
Ru1	0.60457 (10)	0.20821 (3)	−0.00508 (7)	0.0216 (3)	
Cl1	0.72119 (18)	0.23046 (5)	0.19599 (11)	0.0291 (6)	0.399 (11)
Br1	0.72119 (18)	0.23046 (5)	0.19599 (11)	0.0291 (6)	0.601 (11)
Cl2	0.2998 (2)	0.19995 (6)	0.03585 (16)	0.0322 (7)	0.630 (11)
Br2	0.2998 (2)	0.19995 (6)	0.03585 (16)	0.0322 (7)	0.370 (11)
N1	0.6449 (10)	0.2957 (3)	−0.1343 (8)	0.0231 (18)	
N2	0.5256 (11)	0.3219 (3)	0.0063 (7)	0.0247 (18)	
C1	0.5792 (12)	0.2804 (4)	−0.0395 (9)	0.024 (2)	
C2	0.6346 (13)	0.3450 (4)	−0.1471 (9)	0.027 (2)	
C3	0.6940 (14)	0.3753 (4)	−0.2264 (10)	0.030 (2)	
H3	0.7496	0.3630	−0.2868	0.036*	
C4	0.6679 (14)	0.4238 (4)	−0.2125 (9)	0.029 (2)	
H4	0.7055	0.4459	−0.2653	0.035*	
C5	0.5879 (14)	0.4421 (4)	−0.1236 (10)	0.029 (2)	
H5	0.5708	0.4763	−0.1171	0.035*	
C6	0.5345 (15)	0.4115 (4)	−0.0466 (10)	0.032 (2)	
H6	0.4813	0.4241	0.0145	0.038*	
C7	0.5573 (13)	0.3620 (3)	−0.0571 (9)	0.024 (2)	
C8	0.7150 (15)	0.2616 (4)	−0.2085 (9)	0.029 (2)	
H8A	0.8336	0.2705	−0.2176	0.035*	
H8B	0.6427	0.2616	−0.2810	0.035*	
C9	0.7140 (14)	0.2114 (4)	−0.1541 (9)	0.026 (2)	
C10	0.5597 (15)	0.1828 (4)	−0.1780 (10)	0.030 (2)	
H10	0.4690	0.1900	−0.2401	0.036*	
C11	0.5439 (14)	0.1426 (4)	−0.1061 (9)	0.029 (2)	
H11	0.4374	0.1237	−0.1194	0.034*	
C12	0.6761 (13)	0.1292 (4)	−0.0165 (9)	0.029 (2)	
C13	0.8302 (14)	0.1597 (4)	0.0012 (10)	0.030 (2)	
H13	0.9203	0.1524	0.0637	0.036*	
C14	0.8585 (14)	0.1996 (3)	−0.0666 (9)	0.025 (2)	
H14	0.9670	0.2177	−0.0552	0.030*	
C15	0.6573 (14)	0.0866 (4)	0.0614 (9)	0.027 (2)	
C16	0.6828 (15)	0.1024 (4)	0.1864 (10)	0.034 (2)	
H16A	0.8046	0.1110	0.2103	0.052*	
H16B	0.6511	0.0763	0.2317	0.052*	
H16C	0.6086	0.1297	0.1945	0.052*	
C17	0.4716 (15)	0.0640 (4)	0.0349 (10)	0.036 (3)	
H17A	0.3857	0.0858	0.0572	0.054*	
H17B	0.4693	0.0342	0.0752	0.054*	
H17C	0.4440	0.0579	−0.0442	0.054*	
C18	0.7974 (16)	0.0484 (4)	0.0473 (11)	0.038 (3)	
H18A	0.7866	0.0392	−0.0302	0.057*	
H18B	0.7800	0.0206	0.0919	0.057*	
H18C	0.9135	0.0614	0.0714	0.057*	
C19	0.4525 (14)	0.3223 (4)	0.1144 (9)	0.028 (2)	
H19A	0.5491	0.3249	0.1753	0.034*	
H19B	0.3941	0.2922	0.1224	0.034*	
C20	0.3246 (14)	0.3624 (4)	0.1237 (9)	0.025 (2)	
C21	0.1591 (14)	0.3634 (4)	0.0541 (9)	0.030 (2)	
C22	0.0405 (13)	0.4017 (4)	0.0669 (9)	0.027 (2)	
H22	−0.0736	0.4022	0.0205	0.032*	
C23	0.0847 (15)	0.4383 (4)	0.1445 (9)	0.032 (2)	
C24	0.2486 (15)	0.4370 (4)	0.2112 (10)	0.035 (3)	
H24	0.2804	0.4627	0.2641	0.042*	
C25	0.3703 (15)	0.3996 (4)	0.2046 (9)	0.032 (2)	
C26	0.1006 (16)	0.3244 (4)	−0.0281 (11)	0.039 (3)	
H26A	0.1386	0.3318	−0.0985	0.058*	
H26B	−0.0263	0.3220	−0.0387	0.058*	
H26C	0.1517	0.2943	−0.0001	0.058*	
C27	−0.0427 (18)	0.4793 (5)	0.1538 (12)	0.046 (3)	
H27A	−0.0544	0.4841	0.2312	0.068*	
H27B	−0.1563	0.4716	0.1116	0.068*	
H27C	0.0019	0.5083	0.1244	0.068*	
C28	0.5490 (16)	0.4006 (4)	0.2781 (10)	0.037 (3)	
H28A	0.5585	0.4292	0.3235	0.055*	
H28B	0.6408	0.4006	0.2318	0.055*	
H28C	0.5613	0.3727	0.3258	0.055*	

### Atomic displacement parameters (Å^2^)

**Table:**

	*U*^11^	*U*^22^	*U*^33^	*U*^12^	*U*^13^	*U*^23^
Ru1	0.0204 (5)	0.0223 (5)	0.0219 (5)	−0.0002 (3)	0.0021 (3)	−0.0011 (3)
Cl1	0.0329 (8)	0.0257 (8)	0.0261 (8)	0.0028 (5)	−0.0035 (5)	−0.0018 (5)
Br1	0.0329 (8)	0.0257 (8)	0.0261 (8)	0.0028 (5)	−0.0035 (5)	−0.0018 (5)
Cl2	0.0263 (10)	0.0370 (11)	0.0354 (11)	−0.0006 (7)	0.0118 (7)	0.0017 (7)
Br2	0.0263 (10)	0.0370 (11)	0.0354 (11)	−0.0006 (7)	0.0118 (7)	0.0017 (7)
N1	0.016 (4)	0.019 (4)	0.035 (5)	0.000 (3)	0.007 (3)	0.007 (3)
N2	0.026 (4)	0.025 (5)	0.023 (4)	0.009 (3)	0.001 (3)	−0.001 (3)
C1	0.017 (4)	0.030 (5)	0.024 (5)	−0.006 (4)	0.002 (4)	−0.001 (4)
C2	0.023 (5)	0.027 (5)	0.031 (6)	0.000 (4)	0.005 (4)	0.003 (4)
C3	0.029 (5)	0.031 (6)	0.033 (6)	−0.002 (4)	0.011 (5)	0.006 (4)
C4	0.031 (5)	0.034 (6)	0.023 (5)	−0.002 (4)	0.009 (4)	0.004 (4)
C5	0.029 (5)	0.016 (5)	0.040 (6)	0.001 (4)	0.001 (5)	0.005 (4)
C6	0.035 (6)	0.029 (6)	0.034 (6)	0.008 (4)	0.016 (5)	0.005 (5)
C7	0.023 (5)	0.019 (5)	0.031 (5)	0.007 (4)	0.011 (4)	0.009 (4)
C8	0.042 (6)	0.027 (5)	0.021 (5)	−0.001 (4)	0.009 (4)	0.000 (4)
C9	0.030 (5)	0.025 (5)	0.025 (5)	−0.002 (4)	0.014 (4)	0.001 (4)
C10	0.035 (6)	0.025 (5)	0.033 (6)	−0.004 (4)	0.013 (5)	−0.012 (4)
C11	0.030 (5)	0.022 (5)	0.033 (6)	0.004 (4)	0.004 (4)	−0.012 (4)
C12	0.023 (5)	0.032 (6)	0.032 (6)	−0.007 (4)	0.010 (4)	−0.001 (4)
C13	0.025 (5)	0.031 (6)	0.037 (6)	0.004 (4)	0.010 (4)	−0.002 (5)
C14	0.036 (6)	0.017 (5)	0.026 (5)	0.002 (4)	0.021 (4)	−0.001 (4)
C15	0.029 (5)	0.027 (5)	0.024 (5)	0.001 (4)	0.001 (4)	0.000 (4)
C16	0.034 (6)	0.036 (6)	0.033 (6)	−0.006 (5)	0.004 (5)	0.006 (5)
C17	0.033 (6)	0.029 (6)	0.042 (7)	−0.012 (4)	−0.004 (5)	0.010 (5)
C18	0.045 (7)	0.024 (6)	0.045 (7)	0.002 (5)	0.005 (5)	−0.001 (5)
C19	0.026 (5)	0.035 (6)	0.025 (5)	0.004 (4)	0.004 (4)	0.001 (4)
C20	0.034 (5)	0.022 (5)	0.021 (5)	−0.004 (4)	0.004 (4)	−0.002 (4)
C21	0.027 (5)	0.034 (6)	0.028 (6)	−0.007 (4)	−0.001 (4)	0.001 (4)
C22	0.021 (5)	0.029 (5)	0.031 (6)	0.003 (4)	0.002 (4)	0.001 (4)
C23	0.036 (6)	0.029 (6)	0.031 (6)	0.006 (5)	0.010 (5)	−0.005 (5)
C24	0.038 (6)	0.028 (6)	0.040 (7)	−0.006 (5)	0.012 (5)	−0.013 (5)
C25	0.036 (6)	0.036 (6)	0.022 (5)	−0.005 (5)	0.004 (4)	−0.001 (4)
C26	0.032 (6)	0.033 (6)	0.046 (7)	0.009 (5)	−0.012 (5)	−0.015 (5)
C27	0.053 (8)	0.034 (7)	0.051 (8)	0.017 (6)	0.011 (6)	−0.004 (6)
C28	0.038 (6)	0.037 (6)	0.035 (6)	−0.007 (5)	0.005 (5)	−0.011 (5)

### Geometric parameters (Å, °)

**Table:**

Ru1—C1	2.048 (11)	C13—H13	0.9600
Ru1—C9	2.095 (10)	C14—H14	0.9600
Ru1—C10	2.177 (11)	C15—C18	1.533 (15)
Ru1—C13	2.177 (10)	C15—C17	1.538 (14)
Ru1—C14	2.192 (10)	C15—C16	1.553 (15)
Ru1—C11	2.198 (10)	C16—H16A	0.9599
Ru1—C12	2.267 (11)	C16—H16B	0.9599
Ru1—Cl2	2.4613 (18)	C16—H16C	0.9599
Ru1—Cl1	2.5267 (17)	C17—H17A	0.9599
N1—C2	1.376 (13)	C17—H17B	0.9599
N1—C1	1.383 (13)	C17—H17C	0.9599
N1—C8	1.457 (13)	C18—H18A	0.9599
N2—C1	1.366 (13)	C18—H18B	0.9599
N2—C7	1.390 (13)	C18—H18C	0.9599
N2—C19	1.494 (13)	C19—C20	1.495 (14)
C2—C7	1.394 (15)	C19—H19A	0.9600
C2—C3	1.399 (15)	C19—H19B	0.9600
C3—C4	1.372 (16)	C20—C21	1.405 (15)
C3—H3	0.9600	C20—C25	1.425 (15)
C4—C5	1.407 (15)	C21—C22	1.418 (15)
C4—H4	0.9600	C21—C26	1.489 (15)
C5—C6	1.365 (15)	C22—C23	1.387 (15)
C5—H5	0.9600	C22—H22	0.9600
C6—C7	1.392 (15)	C23—C24	1.380 (16)
C6—H6	0.9600	C23—C27	1.511 (15)
C8—C9	1.540 (14)	C24—C25	1.404 (16)
C8—H8A	0.9600	C24—H24	0.9600
C8—H8B	0.9600	C25—C28	1.509 (16)
C9—C10	1.414 (15)	C26—H26A	0.9599
C9—C14	1.442 (16)	C26—H26B	0.9599
C10—C11	1.427 (16)	C26—H26C	0.9599
C10—H10	0.9600	C27—H27A	0.9599
C11—C12	1.410 (15)	C27—H27B	0.9599
C11—H11	0.9600	C27—H27C	0.9599
C12—C13	1.438 (14)	C28—H28A	0.9599
C12—C15	1.529 (14)	C28—H28B	0.9599
C13—C14	1.411 (14)	C28—H28C	0.9599
			
C1—Ru1—C9	79.8 (4)	Ru1—C11—H11	130.2
C1—Ru1—C10	97.3 (4)	C11—C12—C13	115.6 (10)
C9—Ru1—C10	38.6 (4)	C11—C12—C15	123.4 (9)
C1—Ru1—C13	131.3 (4)	C13—C12—C15	121.0 (10)
C9—Ru1—C13	68.8 (4)	C11—C12—Ru1	68.9 (6)
C10—Ru1—C13	80.9 (4)	C13—C12—Ru1	67.8 (6)
C1—Ru1—C14	95.7 (4)	C15—C12—Ru1	131.5 (7)
C9—Ru1—C14	39.2 (4)	C14—C13—C12	124.7 (11)
C10—Ru1—C14	70.0 (4)	C14—C13—Ru1	71.7 (6)
C13—Ru1—C14	37.7 (4)	C12—C13—Ru1	74.6 (6)
C1—Ru1—C11	133.7 (4)	C14—C13—H13	117.7
C9—Ru1—C11	68.8 (4)	C12—C13—H13	117.7
C10—Ru1—C11	38.1 (4)	Ru1—C13—H13	128.7
C13—Ru1—C11	66.9 (4)	C13—C14—C9	115.7 (9)
C14—Ru1—C11	81.1 (4)	C13—C14—Ru1	70.6 (6)
C1—Ru1—C12	161.6 (4)	C9—C14—Ru1	66.8 (6)
C9—Ru1—C12	81.8 (4)	C13—C14—H14	122.1
C10—Ru1—C12	68.5 (4)	C9—C14—H14	122.1
C13—Ru1—C12	37.7 (4)	Ru1—C14—H14	133.1
C14—Ru1—C12	68.9 (3)	C12—C15—C18	109.5 (9)
C11—Ru1—C12	36.8 (4)	C12—C15—C17	110.9 (9)
C1—Ru1—Cl2	93.9 (3)	C18—C15—C17	109.4 (9)
C9—Ru1—Cl2	133.5 (3)	C12—C15—C16	111.6 (9)
C10—Ru1—Cl2	98.4 (3)	C18—C15—C16	108.5 (9)
C13—Ru1—Cl2	134.6 (3)	C17—C15—C16	106.9 (9)
C14—Ru1—Cl2	165.8 (3)	C15—C16—H16A	109.5
C11—Ru1—Cl2	84.7 (3)	C15—C16—H16B	109.5
C12—Ru1—Cl2	99.6 (3)	H16A—C16—H16B	109.5
C1—Ru1—Cl1	87.9 (3)	C15—C16—H16C	109.5
C9—Ru1—Cl1	133.1 (3)	H16A—C16—H16C	109.5
C10—Ru1—Cl1	167.9 (3)	H16B—C16—H16C	109.5
C13—Ru1—Cl1	87.5 (3)	C15—C17—H17A	109.5
C14—Ru1—Cl1	98.7 (3)	C15—C17—H17B	109.5
C11—Ru1—Cl1	138.3 (3)	H17A—C17—H17B	109.5
C12—Ru1—Cl1	104.0 (3)	C15—C17—H17C	109.5
Cl2—Ru1—Cl1	92.04 (7)	H17A—C17—H17C	109.5
C2—N1—C1	112.0 (9)	H17B—C17—H17C	109.5
C2—N1—C8	126.5 (9)	C15—C18—H18A	109.5
C1—N1—C8	121.5 (8)	C15—C18—H18B	109.5
C1—N2—C7	111.3 (8)	H18A—C18—H18B	109.5
C1—N2—C19	122.3 (9)	C15—C18—H18C	109.5
C7—N2—C19	126.4 (8)	H18A—C18—H18C	109.5
N2—C1—N1	104.2 (9)	H18B—C18—H18C	109.5
N2—C1—Ru1	140.1 (8)	N2—C19—C20	114.0 (8)
N1—C1—Ru1	115.6 (7)	N2—C19—H19A	108.8
N1—C2—C7	105.8 (9)	C20—C19—H19A	108.8
N1—C2—C3	130.9 (10)	N2—C19—H19B	108.8
C7—C2—C3	123.3 (10)	C20—C19—H19B	108.8
C4—C3—C2	115.9 (10)	H19A—C19—H19B	107.7
C4—C3—H3	122.1	C21—C20—C25	119.8 (10)
C2—C3—H3	122.1	C21—C20—C19	120.5 (9)
C3—C4—C5	122.4 (10)	C25—C20—C19	119.6 (10)
C3—C4—H4	118.8	C20—C21—C22	118.7 (10)
C5—C4—H4	118.8	C20—C21—C26	122.2 (10)
C6—C5—C4	120.1 (10)	C22—C21—C26	118.9 (9)
C6—C5—H5	119.9	C23—C22—C21	121.8 (10)
C4—C5—H5	119.9	C23—C22—H22	119.1
C5—C6—C7	119.8 (10)	C21—C22—H22	119.1
C5—C6—H6	120.1	C24—C23—C22	118.7 (10)
C7—C6—H6	120.1	C24—C23—C27	120.8 (11)
N2—C7—C6	134.7 (10)	C22—C23—C27	120.5 (11)
N2—C7—C2	106.7 (8)	C23—C24—C25	122.3 (10)
C6—C7—C2	118.5 (9)	C23—C24—H24	118.8
N1—C8—C9	107.4 (8)	C25—C24—H24	118.8
N1—C8—H8A	110.2	C24—C25—C20	118.6 (10)
C9—C8—H8A	110.2	C24—C25—C28	120.0 (10)
N1—C8—H8B	110.2	C20—C25—C28	121.3 (10)
C9—C8—H8B	110.2	C21—C26—H26A	109.5
H8A—C8—H8B	108.5	C21—C26—H26B	109.5
C10—C9—C14	122.8 (9)	H26A—C26—H26B	109.5
C10—C9—C8	118.7 (10)	C21—C26—H26C	109.5
C14—C9—C8	117.6 (9)	H26A—C26—H26C	109.5
C10—C9—Ru1	73.8 (6)	H26B—C26—H26C	109.5
C14—C9—Ru1	74.0 (6)	C23—C27—H27A	109.5
C8—C9—Ru1	115.6 (7)	C23—C27—H27B	109.5
C9—C10—C11	117.3 (11)	H27A—C27—H27B	109.5
C9—C10—Ru1	67.6 (6)	C23—C27—H27C	109.5
C11—C10—Ru1	71.8 (6)	H27A—C27—H27C	109.5
C9—C10—H10	121.3	H27B—C27—H27C	109.5
C11—C10—H10	121.3	C25—C28—H28A	109.5
Ru1—C10—H10	131.9	C25—C28—H28B	109.5
C12—C11—C10	123.6 (10)	H28A—C28—H28B	109.5
C12—C11—Ru1	74.3 (6)	C25—C28—H28C	109.5
C10—C11—Ru1	70.2 (6)	H28A—C28—H28C	109.5
C12—C11—H11	118.2	H28B—C28—H28C	109.5
C10—C11—H11	118.2		
			
C7—N2—C1—N1	−0.3 (11)	C14—Ru1—C11—C10	−68.9 (6)
C19—N2—C1—N1	−177.2 (8)	C12—Ru1—C11—C10	−135.5 (9)
C7—N2—C1—Ru1	174.3 (9)	Cl2—Ru1—C11—C10	110.8 (6)
C19—N2—C1—Ru1	−2.5 (16)	Cl1—Ru1—C11—C10	−162.0 (5)
C2—N1—C1—N2	0.6 (11)	C10—C11—C12—C13	1.7 (15)
C8—N1—C1—N2	−179.5 (9)	Ru1—C11—C12—C13	−50.7 (8)
C2—N1—C1—Ru1	−175.6 (7)	C10—C11—C12—C15	179.1 (9)
C8—N1—C1—Ru1	4.4 (12)	Ru1—C11—C12—C15	126.7 (10)
C9—Ru1—C1—N2	−175.8 (12)	C10—C11—C12—Ru1	52.4 (9)
C10—Ru1—C1—N2	149.7 (11)	C1—Ru1—C12—C11	−68.9 (13)
C13—Ru1—C1—N2	−126.1 (11)	C9—Ru1—C12—C11	−65.3 (7)
C14—Ru1—C1—N2	−139.8 (11)	C10—Ru1—C12—C11	−27.7 (6)
C11—Ru1—C1—N2	137.2 (10)	C13—Ru1—C12—C11	−131.1 (9)
C12—Ru1—C1—N2	−172.2 (10)	C14—Ru1—C12—C11	−103.7 (7)
Cl2—Ru1—C1—N2	50.7 (11)	Cl2—Ru1—C12—C11	67.7 (6)
Cl1—Ru1—C1—N2	−41.3 (11)	Cl1—Ru1—C12—C11	162.2 (6)
C9—Ru1—C1—N1	−1.6 (7)	C1—Ru1—C12—C13	62.2 (14)
C10—Ru1—C1—N1	−36.1 (8)	C9—Ru1—C12—C13	65.8 (7)
C13—Ru1—C1—N1	48.1 (9)	C10—Ru1—C12—C13	103.4 (7)
C14—Ru1—C1—N1	34.4 (8)	C14—Ru1—C12—C13	27.3 (6)
C11—Ru1—C1—N1	−48.6 (9)	C11—Ru1—C12—C13	131.1 (9)
C12—Ru1—C1—N1	2.0 (16)	Cl2—Ru1—C12—C13	−161.3 (6)
Cl2—Ru1—C1—N1	−135.1 (7)	Cl1—Ru1—C12—C13	−66.7 (6)
Cl1—Ru1—C1—N1	133.0 (7)	C1—Ru1—C12—C15	174.6 (10)
C1—N1—C2—C7	−0.6 (11)	C9—Ru1—C12—C15	178.2 (10)
C8—N1—C2—C7	179.4 (9)	C10—Ru1—C12—C15	−144.2 (10)
C1—N1—C2—C3	176.0 (11)	C13—Ru1—C12—C15	112.4 (12)
C8—N1—C2—C3	−3.9 (18)	C14—Ru1—C12—C15	139.7 (10)
N1—C2—C3—C4	−177.7 (11)	C11—Ru1—C12—C15	−116.5 (12)
C7—C2—C3—C4	−1.6 (16)	Cl2—Ru1—C12—C15	−48.9 (9)
C2—C3—C4—C5	0.6 (16)	Cl1—Ru1—C12—C15	45.7 (10)
C3—C4—C5—C6	0.6 (17)	C11—C12—C13—C14	−2.8 (15)
C4—C5—C6—C7	−0.8 (17)	C15—C12—C13—C14	179.8 (9)
C1—N2—C7—C6	−177.5 (12)	Ru1—C12—C13—C14	−54.0 (9)
C19—N2—C7—C6	−0.8 (19)	C11—C12—C13—Ru1	51.2 (8)
C1—N2—C7—C2	0.0 (11)	C15—C12—C13—Ru1	−126.2 (9)
C19—N2—C7—C2	176.6 (9)	C1—Ru1—C13—C14	−22.6 (9)
C5—C6—C7—N2	177.1 (11)	C9—Ru1—C13—C14	30.9 (6)
C5—C6—C7—C2	−0.1 (16)	C10—Ru1—C13—C14	69.1 (7)
N1—C2—C7—N2	0.4 (11)	C11—Ru1—C13—C14	106.1 (7)
C3—C2—C7—N2	−176.5 (10)	C12—Ru1—C13—C14	135.5 (10)
N1—C2—C7—C6	178.3 (10)	Cl2—Ru1—C13—C14	161.9 (5)
C3—C2—C7—C6	1.4 (16)	Cl1—Ru1—C13—C14	−107.7 (6)
C2—N1—C8—C9	175.0 (9)	C1—Ru1—C13—C12	−158.1 (6)
C1—N1—C8—C9	−4.9 (13)	C9—Ru1—C13—C12	−104.6 (7)
N1—C8—C9—C10	88.1 (11)	C10—Ru1—C13—C12	−66.4 (7)
N1—C8—C9—C14	−81.3 (11)	C14—Ru1—C13—C12	−135.5 (10)
N1—C8—C9—Ru1	3.3 (11)	C11—Ru1—C13—C12	−29.4 (6)
C1—Ru1—C9—C10	−115.6 (7)	Cl2—Ru1—C13—C12	26.4 (8)
C13—Ru1—C9—C10	102.2 (7)	Cl1—Ru1—C13—C12	116.8 (6)
C14—Ru1—C9—C10	132.1 (9)	C12—C13—C14—C9	4.6 (15)
C11—Ru1—C9—C10	29.8 (6)	Ru1—C13—C14—C9	−50.7 (8)
C12—Ru1—C9—C10	65.5 (7)	C12—C13—C14—Ru1	55.2 (9)
Cl2—Ru1—C9—C10	−29.9 (8)	C10—C9—C14—C13	−5.5 (14)
Cl1—Ru1—C9—C10	167.0 (5)	C8—C9—C14—C13	163.5 (9)
C1—Ru1—C9—C14	112.3 (6)	Ru1—C9—C14—C13	52.6 (8)
C10—Ru1—C9—C14	−132.1 (9)	C10—C9—C14—Ru1	−58.0 (9)
C13—Ru1—C9—C14	−29.8 (6)	C8—C9—C14—Ru1	110.9 (9)
C11—Ru1—C9—C14	−102.2 (6)	C1—Ru1—C14—C13	163.1 (7)
C12—Ru1—C9—C14	−66.5 (6)	C9—Ru1—C14—C13	−130.7 (9)
Cl2—Ru1—C9—C14	−162.0 (4)	C10—Ru1—C14—C13	−101.2 (7)
Cl1—Ru1—C9—C14	35.0 (7)	C11—Ru1—C14—C13	−63.4 (7)
C1—Ru1—C9—C8	−1.1 (8)	C12—Ru1—C14—C13	−27.3 (6)
C10—Ru1—C9—C8	114.5 (11)	Cl2—Ru1—C14—C13	−64.4 (14)
C13—Ru1—C9—C8	−143.3 (9)	Cl1—Ru1—C14—C13	74.4 (6)
C14—Ru1—C9—C8	−113.4 (10)	C1—Ru1—C14—C9	−66.2 (6)
C11—Ru1—C9—C8	144.3 (9)	C10—Ru1—C14—C9	29.5 (6)
C12—Ru1—C9—C8	−180.0 (8)	C13—Ru1—C14—C9	130.7 (9)
Cl2—Ru1—C9—C8	84.6 (8)	C11—Ru1—C14—C9	67.3 (6)
Cl1—Ru1—C9—C8	−78.5 (9)	C12—Ru1—C14—C9	103.3 (6)
C14—C9—C10—C11	4.6 (15)	Cl2—Ru1—C14—C9	66.3 (13)
C8—C9—C10—C11	−164.3 (9)	Cl1—Ru1—C14—C9	−155.0 (5)
Ru1—C9—C10—C11	−53.5 (8)	C11—C12—C15—C18	115.9 (11)
C14—C9—C10—Ru1	58.1 (9)	C13—C12—C15—C18	−66.9 (13)
C8—C9—C10—Ru1	−110.7 (9)	Ru1—C12—C15—C18	−153.6 (8)
C1—Ru1—C10—C9	63.5 (7)	C11—C12—C15—C17	−4.9 (15)
C13—Ru1—C10—C9	−67.4 (7)	C13—C12—C15—C17	172.3 (10)
C14—Ru1—C10—C9	−30.0 (6)	Ru1—C12—C15—C17	85.6 (11)
C11—Ru1—C10—C9	−131.2 (9)	C11—C12—C15—C16	−124.0 (11)
C12—Ru1—C10—C9	−104.4 (7)	C13—C12—C15—C16	53.2 (13)
Cl2—Ru1—C10—C9	158.6 (6)	Ru1—C12—C15—C16	−33.5 (13)
Cl1—Ru1—C10—C9	−51.6 (17)	C1—N2—C19—C20	−151.5 (9)
C1—Ru1—C10—C11	−165.3 (6)	C7—N2—C19—C20	32.1 (14)
C9—Ru1—C10—C11	131.2 (9)	N2—C19—C20—C21	67.2 (13)
C13—Ru1—C10—C11	63.9 (6)	N2—C19—C20—C25	−113.5 (11)
C14—Ru1—C10—C11	101.3 (7)	C25—C20—C21—C22	−0.3 (15)
C12—Ru1—C10—C11	26.8 (6)	C19—C20—C21—C22	179.0 (9)
Cl2—Ru1—C10—C11	−70.2 (6)	C25—C20—C21—C26	−177.0 (11)
Cl1—Ru1—C10—C11	79.6 (15)	C19—C20—C21—C26	2.3 (16)
C9—C10—C11—C12	−2.7 (15)	C20—C21—C22—C23	1.1 (16)
Ru1—C10—C11—C12	−54.1 (9)	C26—C21—C22—C23	177.9 (11)
C9—C10—C11—Ru1	51.5 (8)	C21—C22—C23—C24	−0.4 (17)
C1—Ru1—C11—C12	155.9 (6)	C21—C22—C23—C27	178.3 (11)
C9—Ru1—C11—C12	105.3 (7)	C22—C23—C24—C25	−1.2 (17)
C10—Ru1—C11—C12	135.5 (9)	C27—C23—C24—C25	−179.8 (11)
C13—Ru1—C11—C12	30.1 (6)	C23—C24—C25—C20	1.9 (17)
C14—Ru1—C11—C12	66.6 (6)	C23—C24—C25—C28	178.8 (11)
Cl2—Ru1—C11—C12	−113.7 (6)	C21—C20—C25—C24	−1.1 (16)
Cl1—Ru1—C11—C12	−26.5 (8)	C19—C20—C25—C24	179.6 (10)
C1—Ru1—C11—C10	20.4 (9)	C21—C20—C25—C28	−178.0 (10)
C9—Ru1—C11—C10	−30.2 (6)	C19—C20—C25—C28	2.7 (15)
C13—Ru1—C11—C10	−105.4 (7)		

### Hydrogen-bond geometry (Å, °)

**Table:**

*D*—H···*A*	*D*—H	H···*A*	*D*···*A*	*D*—H···*A*
C14—H14···Cl2^i^	0.96	2.65	3.406 (11)	135
C16—H16C···Br1	0.96	2.92	3.563 (11)	125
C19—H19A···Br1	0.96	2.92	3.323 (11)	106

Symmetry codes: (i) *x*+1, *y*, *z*.
